# Differential phenotypic and genetic expression of defence compounds in a plant–herbivore interaction along elevation

**DOI:** 10.1098/rsos.160226

**Published:** 2016-09-28

**Authors:** Ana L. Salgado, Tomasz Suchan, Loïc Pellissier, Sergio Rasmann, Anne-Lyse Ducrest, Nadir Alvarez

**Affiliations:** 1 Department of Ecology and Evolution, Biophore building, University of Lausanne, Lausanne, Switzerland; 2 Metapopulation Research Centre, Faculty of Biological and Environmental Sciences, University of Helsinki, Helsinki, Finland; 3 Unit of Ecology and Evolution, Department of Biology, University of Fribourg, Fribourg, Switzerland; 4 Institute of Biology, University of Neuchâtel, Neuchâtel, Switzerland

**Keywords:** cyanogenic glycosides, elevation gradient, gene expression, *Lotus corniculatus*, predation, *Zygaena filipendulae*

## Abstract

Elevation gradients impose large differences in abiotic and biotic conditions over short distances, in turn, likely driving differences in gene expression more than would genetic variation *per se*, as natural selection and drift are less likely to fix alleles at such a narrow spatial scale. As elevation increases, the pressure exerted on plants by herbivores and on arthropod herbivores by predators decreases, and organisms spanning the elevation gradient are thus expected to show lower levels of defence at high elevation. The alternative hypothesis, based on the optimal defence theory, is that defence allocation should be higher in low-resource habitats such as those at high elevation, due to higher costs associated with tissue replacement. In this study, we analyse variation with elevation in (i) defence compound content in the plant *Lotus corniculatus* and (ii) gene expression associated with defence against predators in the specific phytophagous moth, *Zygaena filipendulae*. Both species produce cyanogenic glycosides (CNglcs) such as lotaustralin and linamarin as defence mechanisms, with the moth, in addition, being able to sequester CNglcs from its host plant. Specifically, we tested the assumption that the defence-associated phenotype in plants and the gene expression in the insect herbivore should covary between low- and high-elevation environments. We found that *L. corniculatus* accumulated more CNglcs at high elevation, a result in agreement with the optimal defence theory. By contrast, we found that the levels of expression in the defence genes of *Z. filipendulae* larvae were not related to the CNglc content of their host plant. Overall, expression levels were not correlated with elevation either, with the exception of the *UGT33A1* gene, which showed a marginally significant trend towards higher expression at high elevation when using a simple statistical framework. These results suggest that the defence phenotype of plants against herbivores, and subsequent herbivore sequestration machineries and *de novo* production, are based on a complex network of interactions.

## 1. Introduction

Phenotypes are the result of variation in gene sequence, gene expression and subsequent molecular modifications within a metabolic scheme that varies across populations under distinct environmental pressures [1,2]. Adaptation along the dimensions of the ecological niche [3] can be caused by molecular changes occurring at the genetic [4], epigenetic [5] or transcription [6] levels, the latter being considered as the predominant driver of phenotypic plasticity [7,8]. Ultimately, fixation of adaptive alleles and epigenetic polymorphisms occurs in populations if natural selection is not counterbalanced by gene flow [9]. An increasing number of studies have documented differential gene expression along environmental gradients. For example, phenotypic differences and associated differential gene expression have been observed for genes dealing with cold and hypoxia stress, and haemoglobin polymorphisms [10–12]. However, few studies have investigated traits that are directly related to interactions among organisms along environmental clines [13,14].

Elevation gradients are particularly well suited to investigate variation in gene expression across contrasting biotic and abiotic conditions [15,16]. Following the optimal defence theory [17], the cost of tissue replacement at high elevation should select for higher defence levels, if plants cannot invest in tolerance [18,19]. In this scenario, insect populations that inhabit low-resource habitats, such as high-elevation environments, should have greater defence mechanisms than their counterparts at lower elevations, mainly as a response to the reduced offspring production, shortened reproductive season and decreased fecundity that they must face [15,20]. On the other hand, due to predictable variation in temperature regimes, one of the biotic parameters that varies most with elevation is the metabolic activity of ectothermic animals (which decreases at higher elevation) [15,21–24]. Therefore, it has been postulated that high-elevation plants, due to a reduction in herbivore pressure, should relax their defences [25,26]. Similarly, herbivores should be selected to invest little in defending against predators or parasitoids at high elevation [15]. However, if high-elevation plants contain reduced levels of chemical defences, we could postulate that there would be selection for those herbivores able to re-allocate plant secondary compounds into their own defence to compensate with increased sequestration activity and/or *de novo* production of defence compounds in herbivore arthropods at higher elevation, particularly in specialists [23,24,27,28].

Cyanogenic glycosides (CNglcs) are generally regarded as products of secondary metabolism that act as a defence mechanism in both plants and animals [29]. While toxic to most generalist herbivores [30–32], several specialized insects have evolved the ability to concentrate and store these toxic compounds upon feeding on plants, and use them for defence against their predators, as in the case of *Zygaena* moths [33–36]. Additionally, several Lepidoptera species within the genera *Zygaena* and *Heliconius* [34] have evolved the ability to produce CNglcs *de novo*, using enzymes analogous to those in the plants [37]. *Zygaena* species use two of the most abundant CNglcs, lotaustralin and linamarin, as defence compounds [36,38]. Following the hypothesis of lower predation at high elevation, we may expect a decrease in the production of linamarin and lotaustralin, and therefore lower defence-gene expression, at higher elevation. By contrast, if the predation rate becomes too costly in low-resource environments—as suggested by the optimal defence theory—we should expect an increase in defence-gene expression at higher elevation. Both hypotheses stand for the plants producing linamarin and lotaustralin as defence compounds, such as *Lotus corniculatus*, the host for several *Zygaena* species [39].

Here, we investigated whether: (i) elevation gradients drive variation in the CNglc content of the plant *L. corniculatus*, (ii) whether variation in CNglc production in the plant drives variation in the expression of genes associated with linamarin and lotaustralin production in the moth *Zygaena filipendulae*, a specialist herbivore on *L. corniculatus* and (iii) whether the elevation gradient drives variation in the expression of those defence-associated genes in the moth. We used the elevation gradient of the Swiss Alps, where both *L. corniculatus* and *Z. filipendulae* can be found in a broad range of elevations and habitats (from 300 up to 3000 m a.s.l.) [40,41], and measured CNglc concentrations in *L. corniculatus* and gene expression related to CNglc production in *Z. filipendulae*.

## 2. Material and methods

### 2.1. Study system

We focused on the six-spotted burnet moth *Z. filipendulae* (Lepidoptera) and its preferred host plant, the bird's-foot trefoil *L. corniculatus* (Fabaceae) to examine if defence mechanisms vary along the elevation gradient. The host plant *L. corniculatus* is also attacked by other specialized moths, such as *Syncopacma cinctella* and *Trifurcula subnitidella*, as well as by more than 30 generalist species [39]. As for *Z. filipendulae*, it is a known prey of a large number of parasitoids [42]. In this system, both the host plant and the lepidopteran herbivore produce CNglcs, as mentioned above: while *L. corniculatus* uses CNglcs to deter several generalist herbivores [43], the larvae and adults of *Z. filipendulae* can do the same against generalist predators such as toads and birds [44,45]; *Z. filipendulae* larvae have also evolved the ability to sequester the CNglcs linamarin and lotaustralin from the host plant [46]. It is already known that CNglc concentration in *Z. filipendulae* larvae varies depending on the concentration in the host plant on which they were reared [45]. *De novo* biosynthesis allows the adjustment of overall content, particularly for larvae feeding on low CNglc or acyanogenic host plants [37,45–47]. Genes involved in the biosynthesis of CNglcs in *Z. filipendulae* include *CYP405A2*, *CYP332A3* and *UGT33A1*; the first two genes are part of the cytochrome P450s family (CYP) whose principal role is the assimilation of xenobiotics, and the third gene is part of the UDP glucuronosyltransferase (UGT) enzyme family, which have a role in detoxification of the compounds produced by the P450 enzymes. These genes also function in the physiological regulation of larval development [48–50].

### 2.2. Tissue sampling

Larvae of *Z. filipendulae* were collected in June and July 2014 in the Swiss Alps, from localities at low (less than or equal to 800 m a.s.l.) and high elevation (greater than or equal to 1500 m.a.s.l). A maximum of four individuals was collected from each locality. The caterpillars were identified in the field following Paolucci [51], sorted from first to seventh larval instar according to their size (with an eighth stage being used to categorize pupae), and later DNA barcoded (see below) to confirm their identification. Individuals were cut in half with sterilized scalpel and tweezers. The head, thorax and first three abdominal segments were preserved in 1 ml of 70% ethanol for DNA extraction. The last seven abdominal segments were preserved in 1 ml of RNA*later* RNA Stabilization Reagent (Qiagen) for RNA extraction. Subsequently, the samples were transported to the laboratory and stored at −20°C. Samples of *L. corniculatus* host plants (from the plant on which each larva was found), were collected in envelopes, transported on ice, weighed (five leaves per sample) and stored individually at −80°C in 96-well PCR plates to preserve CNglc content.

### 2.3. CNglc content in *Lotus corniculatus*

CNglc concentration measurements were performed using the Feigl–Anger method following Takos *et al*. [32]. This test allows a semi-quantitative analysis of CNglcs based on the reaction of copper acetate in the presence of cyanide [52]. The test paper was placed on the PCR plate containing the frozen samples and they were then returned to the freezer at −20°C in order to destroy the cell wall and start the cyanogenesis reaction. A first assay confirmed that there was no need to macerate the tissue in order to quantify the cyanide present in the leaf samples. After 1 h of reaction the test papers were digitized and scored using the Dot Blot Analysis function in ImageJ 1.48v software [53]. From each plant, the mean of the IntDen index from five leaves was used. The index corresponds to the Integrated Density of an image, which is calculated from the area and the mean of the grey value of each image or selection [53]. We did not use a reference in order to quantify each sample according to its linamarin/lotaustralin content, but instead used relative index values. Relative quantities of CNglc compounds in plants were corrected for leaf mass, by dividing the mean IntDen index by the mean weight of the five leaves of each host plant.

### 2.4. Barcoding *Zygaena* moths with cytochrome *c* oxidase I

In order to confirm species identification of the larvae, we barcoded 29 sampled specimens from across the sampling locations. DNA extraction of larvae was performed using the DNeasy Blood & Tissue kit (Qiagen, Hombrechtikon, Switzerland). DNA quantification was performed using NanoDrop (Witec, Luzern, Switzerland) and barcoding was performed using cytochrome *c* oxidase I (*COI*) primers (F N2185; R N3014; [54]). Ten nanograms of DNA was used per reaction in 20 µl total volume with 0.25 U Qiagen *Taq* DNA Polymerase, 2 µl buffer 10×, 0.64 µl MgCl_2_ 25 mM, 0.2 µl dNTPs 25 mM and 1 µl primers 10 µM. The PCR was run with the following conditions: 95°C for 1.5 min for denaturation and 35 cycles at 95°C for 35 s, 52°C for 1 min, and 72°C for 1 min, followed by final elongation at 72°C for 8 min. The QIAquick PCR Purification Kit (Qiagen) was used for PCR product purification. Cycle sequencing was carried out with the BigDye® Terminator v. 3.1 Cycle Sequencing Kit (Life Technologies, Zug, Switzerland) with the following conditions: denaturation at 96°C for 2 min, 35 cycles at 96°C for 15 s, 52°C for 15 s and 60°C for 3 s. Sequencing was carried out with both forward and reverse primers, purifying the products using ethanol precipitation and running them on an Automatic Sequencer 3730xl (Applied Biosystems, Foster City, USA). After incorporating sequences of additional *Zygaena* species and the outgroup *Carposina sasakii*, all retrieved from GenBank (see accession numbers in electronic supplementary material, figure S1), sequence alignment was performed using the ClustalW algorithm implemented in Bioedit 7.0 [55], followed by minor manual correction. A phylogenetic tree was generated using the maximum-likelihood algorithm implemented in RAxML [56] run on the CIPRES portal [57]. The analysis was performed using ten alternative runs on distinct starting trees, with the GTR + G substitution model, 25 substitution rate categories and 1000 bootstrap iterations.

### 2.5. Gene expression of defence-associated genes in *Zygaena filipendulae*

For all confirmed *Z. filipendulae* samples, total RNA was extracted with the RNeasy Mini Kit (Qiagen) from 10 mg of moth abdomen. RNA was eluted in 30 µl of RNA-free water and quantified with a Qubit® 2.0 Fluorometer (Life Technologies). Sample quality was assessed with a fragment analyser (Advanced Analytical Technology I, Labgene, Châtel-St-Denis, Switzerland). To avoid genomic DNA contamination, 1 µg of total RNA was treated with 5 U of DNAse I (Roche, Basel, Switzerland) at 37°C for 30 min in a 10 µl solution containing 10 µM Tris–HCl pH 8, 0.5 µM MgCl_2_, 1 mM DTT and 10 U Rnasin Plus RNase Inhibitor (Promega, Dübendorf, Switzerland), followed by enzyme deactivation at 65°C for 10 min. Then 1/10 of the DNAse I treated RNA was reverse transcribed using the SuperScript® III First-Strand Synthesis Kit (Life Technologies), with 50 ng of random hexamer primers (Microsynth, Balgach, Switzerland), 4 U of Rnasin Plus RNase Inhibitor (Promega), 500 µM of dNTP in 20 µl. To avoid qPCR inhibition, cDNAs were purified using ethanol precipitation with 0.5 volume of 5 M NH_4_OAc (pH 8), 2.5 volumes of ethanol 96% and resuspended in 20 µl of 10 mM Tris–HCl (pH 8) and 0.1 mM EDTA. Gene expression was assessed by quantitative real-time PCR (qRT-PCR) for *CYP332A3*, *CYP405A2* and *UGT33A1*. RNA polymerase II (*RPII*), Actine (*ACT*) and glyceraldehyde 3-phosphate dehydrogenase (*GAPDH*) were used as reference genes in order to normalize expression levels. For *UGT33A1* and *RPII* we used the primers and protocol of Fürstenberg-Hägg *et al*. [58]. New primers were designed using Primer3 [59] and PrimerSelect (DNASTAR, Madison, USA) for the amplification of the other genes, and their specificity was checked by BLASTN. The new sequences are:

(i) *ACT* forward 5′-GTA CGA GCT TCC CGA CGG TCA G-3′,
(ii) *ACT* reverse 5′-TAC CGC ACG ACT CCA TAC CCA G-3′,
(iii) *GAPDH* forward 5′-TTC CGT GTT CCA GTC CCC AAT GTT T-3′,
(iv) *GAPDH* reverse 5′-TCC TTC AGC GGC TTC CTT GAC TTT T-3′,
(v) *CYP405A2* forward 5′-GTG ATG CTT TGC GAA CCA GAT GAC A-3′,
(vi) *CYP405A2* reverse 5′-CTT GCG GGT CGA CTT CCA TTT CTC A-3′,
(vii) *CYP332A3* forward 5′-CGA CGA TGT GAC TGT GGA AAA GGG T-3′, and
(viii) *CYP332A3* reverse 5′-GCC ACA CTT CGG GAT CAG AGA ACT C-3′.

More detailed information about the qRT-PCR can be found in the MIQE guidelines (see electronic supplementary material, table S1).

The quantification cycle value (CT) was measured with the Applied Biosystems 7500 real-time PCR System using 10 µl SYBR® Green I master mix (Eurogentec, Liège, Belgium) in a total volume of 20 µl per sample with 2 µl of cDNA. PCR thermal cycler conditions for *CYP405A2*, *CYP333A3*, *ACT* and *GAPDH* began with a hot start stage at 50°C for 2 min, then pre-denaturation at 95°C for 10 min, followed by 40 cycles at 95°C for 15 s, 63.5°C for 30 s and 72°C for 1 min. In the case of *UGT33A1*, conditions were as in Fürstenberg-Hägg *et al*. [58]; for *RPII* the 40 cycles consisted of 95°C for 15 s, 62°C for 30 sec and 72°C for 1 min. To control for primer-dimer formation a dissocation stage was added to all runs. Two replicates per sample were performed, and when the differences between the CT values of the replicates were above 0.3, they were repeated. Primer efficiency for the genes was: *CYP405A2* = 94.98%, *CYP332A3* = 97.03%; *UGT33A1* = 93.38%, *ACT* = 93.32%, *GAPDH* = 97.04% and *RPII* = 103.54%, thus CT values were corrected by the efficiency before normalization. The standard curve method was used for normalization of the data, and qBasePLUS 1.3 (Biogazelle, Zwijnaarde, Belgium) was used to calculate the relative expression of the defence genes.

### 2.6. Statistical analyses of CNglc concentration in *Lotus corniculatus* and differential gene expression in *Zygaena filipendulae*

Differences in CNglc concentration of the host plant samples between low- and high-elevation populations were compared using a general linear model in R CRAN [60]. Constancy in gene expression of the reference and the defence-associated genes across the different instars of *Z. filipendulae* was examined by computing Pearson correlations between expression levels and the larval instar stages. Using the lmer function from the lme4 package [61], we examined the level of gene expression in *Z. filipendulae* as a function of (i) the gene considered, (ii) elevation, and (iii) CNglc content of the plant (all fixed factors). We further performed a simple one-tailed Student's *t*-test between levels of expression at low versus high elevation (i.e. to examine optimal defence theory in a simplified framework without considering the CNglc content of the plants) for each gene separately.

## 3. Results

### 3.1. *Zygaena filipendulae* samples and barcoding

A total of 81 *Zygaena* larvae were collected, with 61 samples coming from elevations less than or equal to 800 m a.s.l., and 20 from elevations greater than or equal to 1500 m a.s.l. Among them, 29 specimens were identified as *Z. filipendulae*, and COI barcoding confirmed that 28 of those were correctly assigned (16 at low and 12 at high elevation; only sample L39 was misidentified and excluded from further analyses; GenBank accessions KX773462-KX773490). COI sequences showed low intra-specific genetic variation (see electronic supplementary material, figure S1). The total number of specimens used in qRT-PCR was reduced to 25 (i.e. 13 for low and 12 for high elevation) after RNA-quality analysis. The larval instars of the 25 samples that passed the RNA-quality threshold are given in electronic supplementary material, table S2, and their collection localities are given in electronic supplementary material, table S3.

### 3.2. CNglc content in *Lotus corniculatus* leaves

**Figure 1.**
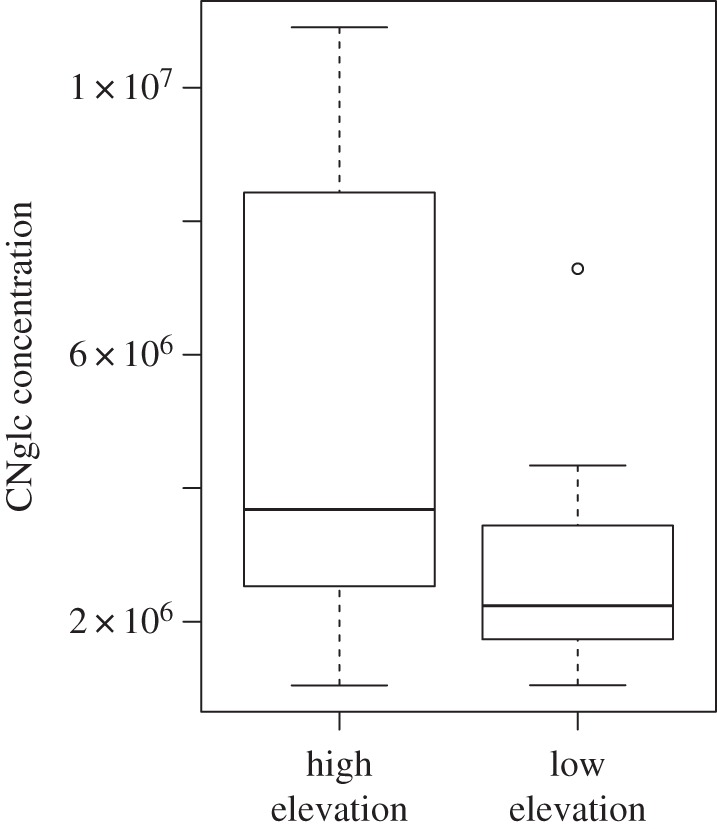
Quantitative analysis of cyanogenic glycoside (CNglc) content in *L. corniculatus* from high- versus low-elevation populations. Low (less than or equal to 800 m a.s.l.; *n* = 13) and high elevation (greater than or equal to 1500 m a.s.l.; *n* = 12) *L. corniculatus* were sampled across the Swiss Alps. CNglcs were quantified as the mean of five replicates of the integrated density value [53] divided by the weight of the leaf for each sample. High-elevation samples contain more CNglcs than low-elevation samples (*p* = 0.0375).

The CNglc content of the host plants where the specimens of *Z. filipendulae* were collected was variable among samples. Our results showed that the CNglc content of the plants differed between high- and low-elevation samples (*F*_1,73_ = 15.48, *p *< 0.01), with higher concentrations in high-elevation (greater than or equal to 1500 m a.s.l.) *L. corniculatus* individuals (figure 1).

### 3.3. Expression of CNglc-associated genes

Measuring the gene expression of CNglc-associated genes in *Z. filipendulae* larvae allowed us to examine differential expression in the synthesis of CNglcs. *RPII*, *ACT* and *GAPDH* were used as reference genes to normalize expression levels because they (i) presented little variation among samples and (ii) did not show a correlation with the larval instar stage of the samples (*ACT*: *F* = 0.01, *p* = 0.90; *GAPDH*: *F* = 0.41, *p* = 0.53; *RPII*: *F* = 0.55, *p* = 0.47; see electronic supplementary material, figure S2). Levels of gene expression for *CYP405A2*, *CYP332A3* and *UGT33A1* did not vary as a function of the larval instar either (*CYP405A2*: *F* = 0.67, *p* = 0.42; *CYP332A3*: *F* = 1.62, *p* = 0.22; *UGT33A1*: *F* = 0.05, *p* = 0.82; see electronic supplementary material, figure S2).

**Figure 2.**
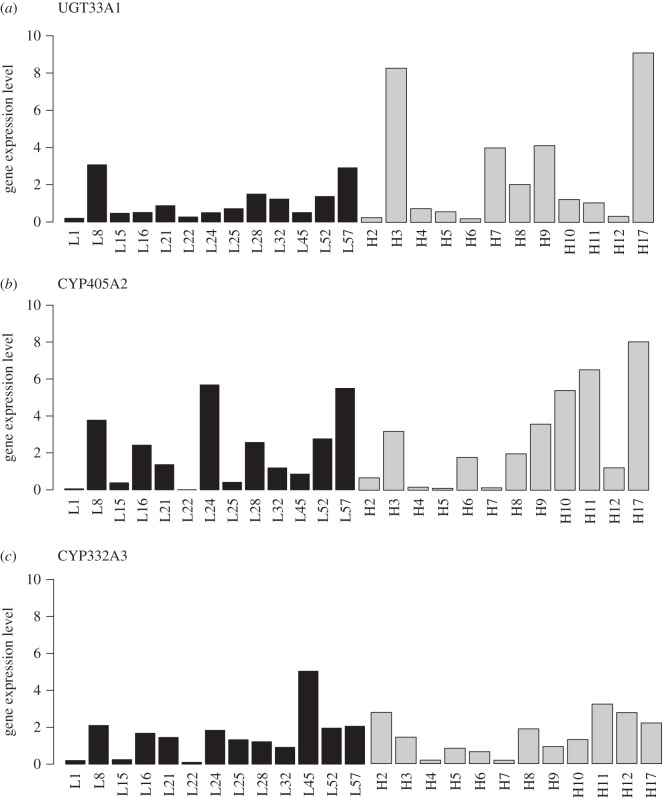
Relative gene expression level of three defence-associated genes in *Z. filipendulae* from low- and high-elevation populations. Samples from low elevation are shown in black (L; less than or equal to 800 m a.s.l.; *n* = 13) and those from high elevation are shown in grey (H; greater than or equal to 1500 m a.s.l.; *n* = 12). Relative expression level in each of the 25 moth samples is given for each of the following genes: *UGT33A1* (*a*; low-elevation mean = 1.087; high-elevation mean = 2.657), *CYP405A2* (*b*; low-elevation mean = 2.070; high-elevation mean = 2.727) and *CYP332A3* (*c*; low-elevation mean = 1.543; high-elevation mean = 1.354). One-tailed Student's *t*-tests revealed a marginally significant higher level of gene expression for *UGT33A1* (*p* = 0.048), but no effect of elevation for *CYP405A2* and *CYP332A3*. However, a more complete linear model showed that none of the genes displayed levels of expression related to elevation or to plant CNglc content (see text).

Our linear model showed that gene expression did not vary among genes (*F* = 0.27, *p* = 0.25) or across elevations (*F* = 2.09, *p* = 0.15), and it was not correlated with the CNglc content of the host plants (*F* = 0.02, *p* = 0.89). Nevertheless, when omitting plant CNglc concentration from the analysis, we found a significant difference between gene expression in moths coming from high versus low elevation for *UGT33A1* (*t* = 1.73, *p *= 0.048), with higher levels in high-elevation samples. Relative expression levels for the three genes are depicted in figure 2.

## 4. Discussion

Our study was performed in order to identify if high- and low-elevation populations are characterized by (i) differential phenotypic expression of defence compounds in *L. corniculatus* and (ii) differential gene expression in defence-associated genes in the specific phytophagous moth *Z. filipendulae*. In addition, we also tested whether gene expression in the moth was correlated with plant CNglc content. In accordance with the resource availability hypothesis [18], we found that high-elevation host plant populations of *L. corniculatus* produced higher levels of CNglcs compared with low-elevation populations. Secondly, we observed no correlation between the CNglc content of host plants and *Z. filipendulae* gene expression, and only a marginal effect of elevation on *Z. filipendulae* populations in the expression of one of the genes involved in CNglc production.

The higher production of CNglcs at high elevation may be linked to limitations on the resources and primary metabolism of plants; in such a situation, allocation to defences should be greater in low-resource (e.g. high elevation) habitats [26]. While, in general, the levels of plant defence compounds should be correlated with herbivore pressure [62,63], and several examples in the literature support this along elevation gradients, an increase in plant defences with elevation is not uncommon [23,24]. For instance, it was recently shown that within the genus *Cardamine*, high-elevation species constitutively produce more glucosinolates, while low-elevation species tend to rely on inducible defences after attack [64]. A potential alternative explanation for our findings is that in addition to providing defence, CNglcs may be involved in other metabolic pathways, such as nitrogen transport and carbon reserves [65,66]. This could result in greater accumulation at high elevation, due to the shorter developmental time that plant populations encounter there. Thus, variation in CNglc content might be uncoupled from levels of herbivory, and emerge as a consequence of other underlying factors, such as climate-driven physiological constraints.

Our second analysis did not show significant differences between low and high populations in the expression of three genes involved in the CNglc production of the moth (except for *UGT33A1* in a simplified statistical framework, see below). The absence of an effect of elevation on the expression of the defence-associated genes could be viewed from the perspective of the plant–insect interaction: if the larvae obtain sufficient CNglcs from the plant—assuming that even if the CNglc content of the plant is lower at low elevation, the concentrations of such compounds are sufficiently high everywhere to fulfil the needs of the moth—there would be no reason to express these genes differently in contrasting environments. An important observation to corroborate our idea is that the larvae of *Z. filipendulae* prefer to feed on high-CNglc plants [47,67]. The pattern observed could also be explained by the disconnection that may occur between the phenotype and the expression of the genes related to this phenotype at a given moment. In this case, once a larva has sequestered or synthesized sufficient CNglcs, it may halt the transcription of genes associated with their production. Indeed, one could imagine that before being collected, the larvae had sequestered or produced enough CNglcs and stopped the transcription of genes associated with CNglc production, whereas its body CNglc concentration is actually high—this point may be particularly relevant given that moths were shown to prefer feeding on high-CNglc content plants, as sequestering defence compounds seems less costly than producing them *de novo* [67]. We could test for this hypothesis in future trials, by directly measuring the level of CNglcs in the larvae at the moment of collection. Nonetheless, when analysing CNglc data in a simplified statistical framework (i.e. with a one-tailed Student's *t*-test), the expression of *UGT33A1* was found to be marginally higher at high versus low elevation (figure 2). Such marginally significant higher amounts of expression at high elevation may indicate, instead, that the optimal defence theory could also be at work here—i.e. as in other *Zygaena* species [68], eggs are laid in batches, making possible the application of the optimal defence theory when incorporating the concept of kin selection. However, one should note that UDP-glucose glycosyltransferase might also be involved in metabolic functions other than the production of defence compounds, as for instance in the regulation of endobiotics [49]. In plants, UDP-glucose glycosyltransferase has also shown to be involved in oxidative stress tolerance [69], a feature that, if it were also occurring in insects, could explain the higher levels of gene expression in moth specimens collected at higher elevations, where UV radiation may increase oxidative stress [70]. Unambiguously identifying whether or not *UGT33A1* shows higher expression at higher elevations would anyway require an increased sample size, as our analyses are based on only 25 observations (13 and 12 at low and high elevation, respectively), meaning that our statistical power is limited. We cannot exclude the possibility that other abiotic factors, such as availability of resources, precipitation and radiation [15], or other molecular mechanisms such as priming [71,72], epigenetic modifications [73] or post-transcriptional effects [74,75] are influencing the expression of the genes involved in the production of CNglcs in *Z. filipendulae.* Finally, future research should also examine the effect of elevation on CNglc-associated gene expression in the plant, in order to gain a more complete picture of the ecological and evolutionary drivers in this plant–herbivore interaction.

## Supplementary Material

Supplementary figure S1. Phylogenetic tree based on COI barcoding of Z. filipendulae samples collected in the Swiss Alps.

## Supplementary Material

Supplementary figure S2. Relative expression of reference and defense-associated genes across different larval instars of Z. filipendulae.

## Supplementary Material

Supplementary table S1. Minimum Information for Publication of Quantitative Real-Time PCR Experiments guideline.

## Supplementary Material

Supplementary table S2. Samples of Z. filipendulae according to their instar.

## Supplementary Material

Supplementary table S3. Localities in the Swiss Alps where samples were collected for Zygaena filipendulae and its host plant Lotus corniculatus.

## Data accessibility

COI sequences are available as GenBank accessions KX773462-KX773490.
