# Supplementary material for: Differential phenotypic and genetic expression of defence compounds in a plant–herbivore interaction along elevation
Source: R Soc Open Sci. 2016 Sep 28;3(9):160226. doi: 10.1098/rsos.160226 (PMC5043307; doi:10.1098/rsos.160226)
Supplement: Supplementary figure S1. Phylogenetic tree based on COI barcoding of Z. filipendulae samples collected in the Swiss Alps. [file rsos160226supp1.docx]

Supplementary figure S1. Phylogenetic tree based on COI barcoding of *Z. filipendulae* samples collected in the Swiss Alps. The analysis includes additional GenBank sequences of *Zygaena* species, as well as one sample from *Carposina sasakii*, which serves as an outgroup. Among the 29 larvae identified in the field as *Z. filipendulae* (in bold), 28 are clustered in the *Z. filipendulae* clade: 16 specimens labelled with an L and 12 specimens labelled with an H, corresponding to low and high elevation samples, respectively, were chosen for further RNA extraction and quality check.
