# Supplementary material for: Differential phenotypic and genetic expression of defence compounds in a plant–herbivore interaction along elevation
Source: R Soc Open Sci. 2016 Sep 28;3(9):160226. doi: 10.1098/rsos.160226 (PMC5043307; doi:10.1098/rsos.160226)
Supplement: Supplementary figure S2. Relative expression of reference and defense-associated genes across different larval instars of Z. filipendulae. [file rsos160226supp2.docx]

Supplementary figure S2. Relative expression of reference and defense-associated genes across different larval instars of *Z. filipendulae*. No relationship is found between the larval stage and the relative expression of any of the genes measured in this study (*ACT*: F = 0.01, *P* = 0.90; *GAPDH*: F = 0.41, *P* = 0.53; *RPII*: F = 0.55, *P* = 0.47; *CYP405A2*: F = 0.67, P = 0.42; *CYP332A3*: F = 1.62, P = 0.22; *UGT33A1*: F = 0.05, P = 0.82). Pearson correlation (r) between the expression level and the larval instar stage is shown on each graph.
