# Supplementary material for: Differential phenotypic and genetic expression of defence compounds in a plant–herbivore interaction along elevation
Source: R Soc Open Sci. 2016 Sep 28;3(9):160226. doi: 10.1098/rsos.160226 (PMC5043307; doi:10.1098/rsos.160226)
Supplement: Supplementary table S1. Minimum Information for Publication of Quantitative Real-Time PCR Experiments guideline. [file rsos160226supp3.docx]

Supplementary table S1. Minimum Information for Publication of Quantitative Real-Time PCR Experiments guideline (MIQE). The aim of the experiment is to determine whether there are differences in the expression of defence-associated genes in *Zygaena filipendulae* larvae that were collected from low and high elevations in the Swiss Alps.

| **Experimental Design** | |
| --- | --- |
| Definition of experimental and control group | Caterpillars of different instars of *Z. filipendulae* collected from 12 different localities in the Swiss Alps. They were placed into two groups for comparison: low elevation (≤800 m.a.s.l.) and high elevation (≥1500 m.a.s.l.). |
| Number within each group | Low elevation = 13; high elevation = 12 |
| **Sample** | |
| Processing procedure | The larvae were cut in half. The last 7 abdominal segments were used for RNA extraction. The head, thorax and the three first abdominal segments were used for DNA extraction. Larvae were alive up to the cutting, immediately followed by RNA*later* storage. |
| Sample storage conditions and duration | The samples were stored in RNA*later* (Qiagen, Hombrechtikon, Switzerland) and stored for 3 months before extraction. |
| **Nucleic Acid Extraction** | |
| Procedure and/or instrumentation | Individual sample was homogenized using pestle in liquid nitrogen. For total RNA extraction, the samples were resuspended in 600 *u*L of RLT buffer containing 6 ul of beta-mercaptoethanol of RNeasy mini kit and further homogeneized with QIAshredder homogenizer (Qiagen, Hombrechtikon, Switzerland) and processed in columns following the manufacturer’s protocol. Total RNA was eluted in 30 *u*L of RNase free water and stored immediately at -80ºC. |
| Name of kit and details of any modification | RNeasy Mini Kit (Qiagen, Hombrechtikon, Switzerland). No modifications. |
| Details of DNase or RNAse treatment | 1 μg of total RNA was treated with 5 U of DNAse I (Roche, Basel, Switzerland) at 37°C for 30 min in 10 μL solution containing 10 μM Tris HCl pH 8, 0.5 μM MgCl_2_, 1 mM DTT and 10 U Rnasin Plus RNase Inhibitor (Promega, Dübendorf, Switzerland), followed by enzyme deactivation at 65°C for 10 min. |
| Contamination Assessment (DNA or RNA) | For each sample, a reverse transcription without the SuperScript® III First-Strand Synthesis Kit (Life Technologies Zug, Switzerland) were prepared and used to check for genomic DNA contamination (RT-) and each RNA was passed on the fragment analyser (see below). |
| Nucleid acid quantification | Qubit® 2.0 Fluorometer (Life Technologies, Zug, Switzerland) with RNA high sensitivity kit. |
| RNA integrity method/instrument | On the fragment analyser, the RQN and 28S/18S ratio were used to determine the integrity of the samples (Advanced Analytical Technology I, Labgene, Châtel-St-Denis, Switzerland). |
| **Reverse transcription** | |
| Complete reaction conditions, reverse transcriptase and concentrations | 100 ng of DNAse I treated RNA was incubated with 50 ng of Random hexamer primers (Microsynth, Balgach, Switzerland), 500 uM of dNTPs at 65°C for 5 min and immediately transferred to 4°C. Then reverse transcribed with 200U of SuperScript® III First-Strand Synthesis Kit (Life Technologies Zug, Switzerland), 40 U of Rnasin Plus RNase Inhibitor ( Promega, Dübendorf, Switzerland ), 5 mM DTT, 1x first strand buffer at 25ºC for 5 min, 50ºC one hour and inactivated at 65ºC for 15 min and stored at -20°C.  Then the 20 ul of cDNA were ethanol precipitated with 10 ul of 5 M NH4OAc (pH 8), 50 ul of cold ethanol 96% and resuspended in 20 ul of 10 mM Tris-HCl (pH 8), 0.1 mM EDTA. |
| Amount of RNA and reaction volume | 100 ng of total RNA was reverse transcribed in 20 *u*L total reaction volume |
| Temperature and time | See complete reaction conditions  (above) |
| Manufacturer of reagents | Microsynth (Balgach, Switzerland) for random hexamer primers. Promega (Dübendorf, Switzerland) for Rnasin Plus RNase Inhibitor and Life Technologies (Zug, Switzerland) for Superscript III RT. |
| Storage conditions of cDNA | Frozen at -20ºC |
| **qPCR Target Information** (For more information see Fürstenberg-Hägg et al. (2014b)) | |
| Amplicon length | UGT33A1: 142; CYP405A2: 150; CYP332A3: 95; ACT: 104; GAPDH: 108; RpII40: 160 bp |
| **qPCR Oligonucleotides** | |
| Primer sequences | CYP405A2 F: 5’ GTGATGCTTTGCGAACCAGATGACA 3’  CYP405A2 R: 5’ CTTGCGGGTCGACTTCCATTTCTCA 3’  CYP332A3 F: 5’ CGACGATGTGACTGTGGAAAAGGGT 3’  CYP332A3 R: 5’ GCCACACTTCGGGATCAGAGAACTC 3’  UGT33A1 F: 5’ AAGCACCACCGAATATCACC 3’  UGT33A1 R: 5’ TAGTGGCCGAACAAAGAACC 3’  ACT F: 5’ GTACGAGCTTCCCGACGGTCAG 3’  ACT R: 5’ TACCGCACGACTCCATACCCAG 3’  GAPDH F: 5’ TTCCGTGTTCCAGTCCCCAATGTTT 3’  GAPDH R: 5’ TCCTTCAGCGGCTTCCTTGACTTTT 3’  RpII40 F: 5’ TCTGGGGATGATGTTGTTATTGG 3’  RpII40 R: 5’ AGATGCGTCACGTTTTGAGAATC 3’ |
| Location and identity of any modification | No modifications |
| Manufacturer of oligonucleotides | Microsynth (Balgach, Switzerland) |
| Purification method | Desalted |
| **qPCR Protocol** | |
| Complete reaction conditions | One master mix was prepared for each primer pairs using 10 ul of 2x qRT-PCR SYBR® Green I (Eurogentec, Liège, Belgium), for primer pair concentration (see below) and water to 18 ul and 2 ul of precipitated cDNA. All the samples were run in duplicate and when the Delta CT value difference was above 0.3, the qPCR was repeated with control samples. For the qPCR condition, see below. A dissociation curve was added at the end of each qPCR |
| Reaction volume and amount of cDNA/DNA | 2 *u*L of cDNA in 20 *u*L total reaction volume |
| Additives (SYBR Green I, DMSO, etc.) | qRT-PCR SYBR® Green I (Eurogentec, Liège, Belgium) |
| Complete thermocycling conditions | Conditions for **UGT33A1**: 50ºC for 2 min, 95ºC for 10 min, followed by 40 cycles at 95ºC for 15 sec, 55ºC 30 sec, 65ºC 30 sec, 78ºC for 45 sec. Dissociation stage at 95ºC for 15 sec, 60ºC for 1 min and 95ºC for 15 sec.  Conditions for **RpII40**: 50ºC for 2 min, 95ºC for 10 min, 40 cycles of 95ºC for 15 sec, 62ºC for 30 sec and 72ºC for 1 min.  Conditions for **CYP405A2, CYP332A3, ACT, GAPDH**:  50ºC for 2 min, 95ºC for 10 min, 40 cycles at 95ºC for 15 sec, 63.5ºC for 30 sec and 72ºC for 1 min. Dissociation stage the same for all genes as described above. |
| Reaction setup (manual/robotic) | Manual |
| Manufacturer of qPCR instrument | Applied Biosystems 7500 Real Time PCR System (Life Technologies Zug, Switzerland) |
| **qPCR validation** | |
| Specificity (gel, sequence, melt or digest) | Amplification of the target genes was verified in 2% agarose gel from the PCR product. « No template control » (NTC) were used in each plate for each gene in qRT-PCR and RT- for each individual (see above). |
| For SYBR Green I, Cq of the NTC | Undetermined |
| Standard curves with slopes and y-intercept | UGT33A1: y = -3.4915x + 29.752; CYP405A2: y = -3.4482x + 31.507; CYP332A3: y = -3.255x + 30.479; RpII40: y = -3.2397+ 35.85; ACT: y = -3.493 + 32.086; GAPDH: y = -3.395x + 31.188. |
| PCR efficiency calculated from slope | UGT33A1: 93.38%; CYP405A2: 94.99%, CYP332A3: 97.03%, ACT: 93.32%, GAPDH: 97.04%; RpII40: 103.54% |
| R2 of standard curve | UGT33A1: 0.99956; CYP405A2: 0.99534, CYP332A3: 0.99633, ACT: 0.99985, GAPDH: 0.99985; RpII40: 0.99784 |
| Evidence for limit of detention | 1/10 serial dilutions for standard curves. |
| **Data Analysis** | |
| qPCR analysis program (source, version) | 7500 System SDS Software (Applied biosystems, Version 1.3.1) |
| Cq method determination | Manual detection of the threshold with an automatic base line |
| Outlier identification and disposition | Cq values with a standard deviation between duplicates equal or greater than 0.30 |
| Repeatability (intra-assay variation) | Standard curve between duplicates equal or less than 0.2 |
| Power analysis | 10 log |
| Statistical methods for results significance | Calibrated normalized relative quantities value (CNRQ) |
| Software (source, version) | qBasePlus (Biogazelle, Version 2.4) |
