# Supplementary material for: Differential phenotypic and genetic expression of defence compounds in a plant–herbivore interaction along elevation
Source: R Soc Open Sci. 2016 Sep 28;3(9):160226. doi: 10.1098/rsos.160226 (PMC5043307; doi:10.1098/rsos.160226)
Supplement: Supplementary table S2. Samples of Z. filipendulae according to their instar. [file rsos160226supp4.docx]

Supplementary table S2. Samples of *Z. filipendulae* according to their instar (instar could not be properly assessed for samples L45, H9, H12 and H17). Stage 8 was used to categorize pupae.

| **Instar** | **Sample ID** | **Total** |
| --- | --- | --- |

| 2 | L8, L16, L22, L52, H3, H11 | 6 |
| --- | --- | --- |
| 3 | L1, H6 | 2 |
| 4 | L15, H2, H8, H10 | 4 |
| 5 | L21, L24, L28, L32 | 6 |
| 6 | L57, H4, H5 | 3 |
| 7 | L25, | 1 |
| 8 | H7 | 1 |
