# Supplementary material for: Differential phenotypic and genetic expression of defence compounds in a plant–herbivore interaction along elevation
Source: R Soc Open Sci. 2016 Sep 28;3(9):160226. doi: 10.1098/rsos.160226 (PMC5043307; doi:10.1098/rsos.160226)
Supplement: Supplementary table S3. Localities in the Swiss Alps where samples were collected for Zygaena filipendulae and its host plant Lotus corniculatus. [file rsos160226supp5.docx]

Supplementary table S3. Localities in the Swiss Alps where samples were collected for *Zygaena filipendulae* and its host plant *Lotus corniculatus.* A total of 25 samples were used for the qRT-PCR of defense-associated genes after barcoding identification and RNA quality determination [“L”, low elevation: collected ≤ 800 m.a.s.l. (n = 13); “H”, high elevation: collected ≥ 1500 m.a.s.l. (n = 12)].

| **Collection number** | **Site** | **Latitude (CH1903)** | **Longitude (CH1903)** | **Elevation (m.a.s.l)** |
| --- | --- | --- | --- | --- |
| L1 | Hérémence | 46178616 | 7415657 | 777 |
| L8 | Evionnaz | 46173945 | 7022266 | 450 |
| L15 | Evionnaz | 46173001 | 7025866 | 463 |
| L16 | Evionnaz | 46173021 | 702587 | 447 |
| L21 | Leuk | 46303813 | 7621003 | 653 |
| L22 | Leuk | 46305241 | 7621917 | 656 |
| L24 | Fully | 46124814 | 7084614 | 468 |
| L25 | Fully | 46124424 | 7084177 | 463 |
| L28 | Fully | 46124962 | 7085028 | 464 |
| L32 | Fully | 46124398 | 7084261 | 466 |
| L45 | Vogelles | 46081795 | 7177452 | 800 |
| L52 | Vispertal | 46255358 | 7878557 | 680 |
| L57 | Vispertal | 46256811 | 7879095 | 730 |
| H2 | Mayen | 4636738 | 7014887 | 1829 |
| H3 | Mayen | 46367385 | 7014838 | 1829 |
| H4 | Mayen | 46367431 | 7014783 | 1825 |
| H5 | Mayen | 46367421 | 701477 | 1824 |
| H6 | Mayen | 46367527 | 7014914 | 1833 |
| H7 | Hochmatt | 46568399 | 7213985 | 1937 |
| H8 | Dessous-La Premiere | 46374019 | 7125176 | 2000 |
| H9 | Dessous-La Premiere | 46366543 | 7120676 | 1568 |
| H10 | Tseseri | 46294014 | 7153184 | 1735 |
| H11 | Bonatchiesse | 46021686 | 7324797 | 1597 |
| H12 | Rochers de Nayes | 46426791 | 6969833 | 1766 |
| H17 | Tseseri | 46294109 | 7153055 | 1737 |
